# A multi-scale modelling framework combining musculoskeletal rigid-body simulations with adaptive finite element analyses, to evaluate the impact of femoral geometry on hip joint contact forces and femoral bone growth

**DOI:** 10.1371/journal.pone.0235966

**Published:** 2020-07-23

**Authors:** Hans Kainz, Bryce Adrian Killen, Mariska Wesseling, Fernando Perez-Boerema, Lorenzo Pitto, Jose Manuel Garcia Aznar, Sandra Shefelbine, Ilse Jonkers

**Affiliations:** 1 Department of Biomechanics, Kinesiology and Computer Science in Sport, University of Vienna, Vienna, Austria; 2 Human Movement Biomechanics Research Group, KU Leuven, Leuven, Belgium; 3 Department of Mechanical Engineering, KU Leuven, Leuven, Belgium; 4 Department of Mechanical Engineering, University of Zaragoza, Zaragoza, Spain; 5 Department of Bioengineering, Northeastern University, Boston, Massachusetts, United States of America; Texas A&M University, UNITED STATES

## Abstract

Multi-scale simulations, combining muscle and joint contact force (JCF) from musculoskeletal simulations with adaptive mechanobiological finite element analysis, allow to estimate musculoskeletal loading and predict femoral growth in children. Generic linearly scaled musculoskeletal models are commonly used. This approach, however, neglects subject- and age-specific musculoskeletal geometry, e.g. femoral neck-shaft angle (NSA) and anteversion angle (AVA). This study aimed to evaluate the impact of proximal femoral geometry, i.e. altered NSA and AVA, on hip JCF and femoral growth simulations. Musculoskeletal models with NSA ranging from 120° to 150° and AVA ranging from 20° to 50° were created and used to calculate muscle and hip JCF based on the gait analysis data of a typically developing child. A finite element model of a paediatric femur was created from magnetic resonance images. The finite element model was morphed to the geometries of the different musculoskeletal models and used for mechanobiological finite element analysis to predict femoral growth trends. Our findings showed that hip JCF increase with increasing NSA and AVA. Furthermore, the orientation of the hip JCF followed the orientation of the femoral neck axis. Consequently, the osteogenic index, which is a function of cartilage stresses and defines the growth rate, barely changed with altered NSA and AVA. Nevertheless, growth predictions were sensitive to the femoral geometry due to changes in the predicted growth directions. Altered NSA had a bigger impact on the growth results than altered AVA. Growth simulations based on mechanobiological principles were in agreement with reported changes in paediatric populations.

## Introduction

Musculoskeletal simulations have been used to examine musculoskeletal loading in paediatric and pathological populations [1–3]. Typically generic musculoskeletal models developed from cadaveric data of an adult are scaled to the anthropometry of the child [4–6]. This procedure neglects subject-specific musculoskeletal geometry, e.g., subject and age-specific femoral neck-shaft angle and anteversion angle [7]. To overcome these limitations, patient-specific musculoskeletal models can be generated from medical images of the participants [8–11]. A small number of studies have compared generic scaled with medical imaging-based models. These studies reported differences in muscle moment arms [12,13], hip joint contact force orientation [14] and joint kinematics [11] between both modelling approaches.

A multi-scale modelling approach, combining muscle and joint contact force estimates from musculoskeletal simulations with adaptive mechanobiological finite element (FE) analysis, can be used to predict femoral growth trends [15–17]. Carriero et al. [15] found a decrease in neck-shaft angle (NSA) and slight increase in anteversion angle (AVA) when modelling femoral growth in one typically developing child. Their study, however, included a musculoskeletal model and adaptive finite element model based on a generic adult model and, therefore, did not consider age- or subject-specific musculoskeletal geometry. Yadav et al. [16] simulated femoral growth in one typically developing child and found a decrease in NSA and AVA when using a FE model based on medical images of the child.

Multi-scale mechanobiological femoral growth simulations have so far only been applied to small samples (n = 1–4) [15–17]. To investigate clinically relevant questions, e.g., if early clinical intervention can be used to avoid the development of femoral deformities in children with cerebral palsy, it is essential to include a larger sample size. In a clinical context, collecting the necessary data (e.g., magnetic resonance images) and generating fully subject-specific models for the femoral growth simulations is rarely possible due to the lack of resources (i.e., time, money, knowledge, limited attention span and tolerance of children). Modifying a generic musculoskeletal and FE model based on average age-specific NSA and AVA would allow model creation and growth simulation execution in a time and cost-efficient manner. However, before this workflow can be used to investigate clinically relevant questions, it is essential to know if the multi-scale modelling workflow and calculated bone growth are sensitive to the musculoskeletal geometry, i.e., for different NSA and AVA.

Previous research showed that subject-specific geometry changes the hip joint contact force orientation [14]. However, no previous studies investigated the impact of femoral geometry on hip joint contact forces (which have the biggest impact on proximal femoral growth simulations [18]) and femoral growth simulations in a systematic way. Hence, the aim of this study was to create musculoskeletal and FE models with a variety of NSA and AVA to evaluate the impact of femoral geometry on hip joint contact force estimations and proximal femoral growth simulations. Based on previous research [19,20], we hypothesized that increased NSA and AVA would lead to increased hip joint contact forces. Furthermore, based on the assumption that musculoskeletal geometry would adapt under aberrant loading conditions, we hypothesized that the increased hip joint contact forces would alter femoral growth simulations.

## Methods

### Participants

Motion capture data of one typically developing child (TD01, 9 years old, weight: 30.4 kg, height: 1.39 m) was analysed for this study. A reference FE model was created based on magnetic resonance images (MRI) collected from a typically developing child (TD02, 8 years old, weight: 20.4 kg, height: 1.24 m, right NSA: 127°, right AVA: 27°). A parent of each child signed informed consent and ethical approval was obtained from the local ethics committee (S57749, Ethical commission UZ/KU Leuven, Belgium).

### Motion capture

The Vicon Plug-in-Gait lower limb marker set [21] with additional three marker clusters on the thighs and shanks and additional six markers on the torso (clavicular, sternum, C7, T10, left and right shoulder) were placed on the child. Marker trajectories and ground reaction forces of one static and several walking trials at a self-selected walking speed were collected with an eight camera motion capture system (Vicon Motion Systems, Oxford, UK) and two force plates (AMTI, Watertown, MA, USA). Vicon Nexus (Vicon Motion Systems, Oxford, UK) was used to label and filter marker trajectories and filter force plate data, with filters being a Butterworth 4th order zero-lag dual-pass, low pass filter with a cut-off frequency of 6 Hz.

### MRI acquisition

MRI were collected using 1.5 T magnetic resonance scanner (MAGNETOM Avanto, Siemens, Berlin/Munic, Germany). A full lower-body scan from the level of above illiac crests to below the toes were obtained in a supine position. The MRI sequence (3D PD SPACE sequence) utilised a slice thickness of 1.1 mm, slice increments of 1.1 mm and a voxel size of 0.8x0.8x1.0 mm [10].

### Musculoskeletal models and simulations

A generic musculoskeletal SIMM (Motion Analysis Corp., Santa Rosa, CA) model [22] with 19 degrees of freedom (DoF) and 88 muscles was scaled to the anthropometry of the child based on the marker locations from the static trial [23]. In this model, the pelvis included six DoF, the hip and pelvis-torso joint included three rotational DoF, and the knee and ankle joint included one DoF in the sagittal plane. After the scaled model was created, the deform tool in SIMM [24,20] was used to create seven models with varying NSA and AVA (Table 1). The deform tool changed the vertices of the femur based on pre-defined boxes to match the chosen NSA and AVA. This procedure alters all the muscle origin and insertion points within the boxes. A detailed description of the deform tool was published previously [24,25].

**Table 1:** Neck-shaft angle and anteversion angle of the seven musculoskeletal model.

Model name	Neck-shaft angle (NSA)	Anteversion angle (AVA)
NSA-120-AVA-20*	**120**	**20**
NSA-120-AVA-30	120	**30**
NSA-120-AVA-40	120	**40**
NSA-120-AVA-50	120	**50**
NSA-130-AVA-20	**130**	20
NSA-140-AVA-20	**140**	20
NSA-150-AVA-20	**150**	20

*reference values for an average adult femoral geometry. The NSA-120-AVA-20 model was used as a reference model for all comparisons (explained in the *data analysis* section).

During typical growth, the NSA decreases from approximately 150° at birth to 120° at skeletal maturity and the AVA decreases from approximately 50° to 20° [7,26,27]. In many children with cerebral palsy the NSA and AVA decreases 10° to 20° less compared to typically developing children [7]. Hence, the created models include a wide range of NSA and AVA, including values from normal adults, typically developing children and children with pathological femoral geometries. The deform tool modified muscle origin and insertion points and, therefore, altered muscle lengths and paths in the models. A Matlab script was used to convert the seven models to an OpenSim model format. Maximum isometric muscle force (MIMF) of the generic model was scaled to the subject’s body weight and multiplied by a scale factor of 1.5 to obtain realistic muscle activations using a customized Matlab script [28,29].

OpenSim 3.3 [4] was used to calculate joint angles, joint moments, muscle and joint contact forces (JCF). Joint angles and moments were calculated using the Kalman smoothing algorithm [30] and inverse dynamics, respectively. Muscle forces were estimated using static optimization, minimizing the sum of squared muscle activations, which is one of the most common ways to calculate muscle forces in OpenSim [2,4,31]. Afterwards, JCF were estimated using OpenSim’s joint reaction analysis [3]. Muscle forces acting on the femur and hip JCF were then used as input for the mechanobiological growth simulations.

### Finite element (FE) model

**Fig 1:**
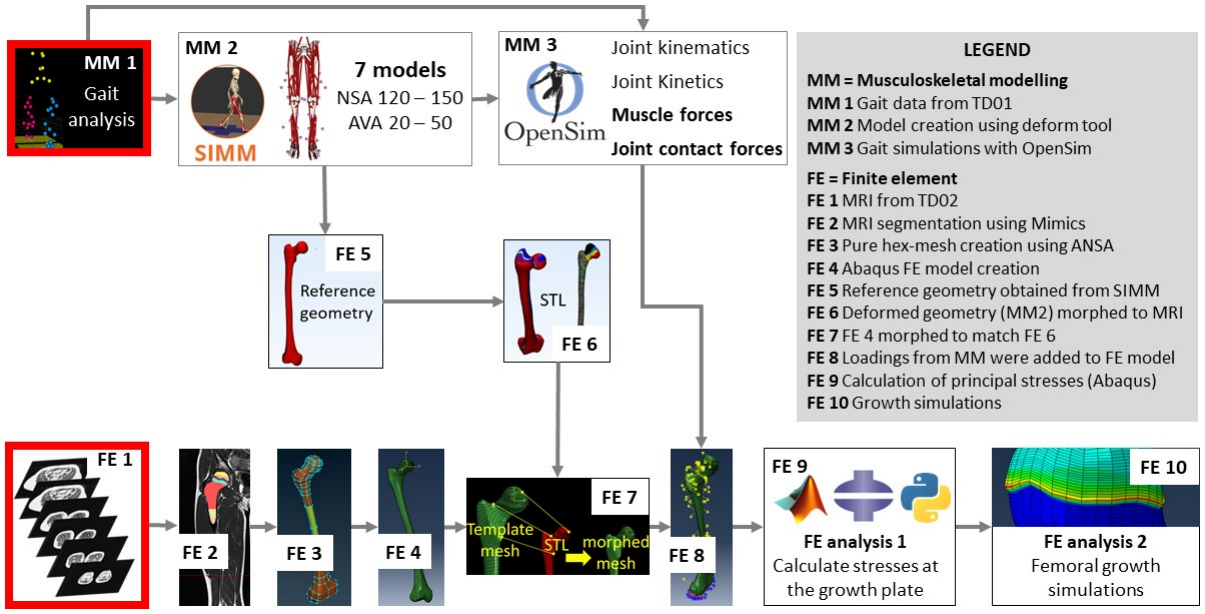
Overview of the workflow to create the finite element model and perform mechanobiological growth simulations. NSA = neck-shaft angle; AVA = anteversion angle; TD = typically developing. Red boxes indicate the input data, i.e., collected motion capture data and magnetic resonance images (MRI). Each step of this workflow is described in detail in the method section of the manuscript.

**Fig 2:**
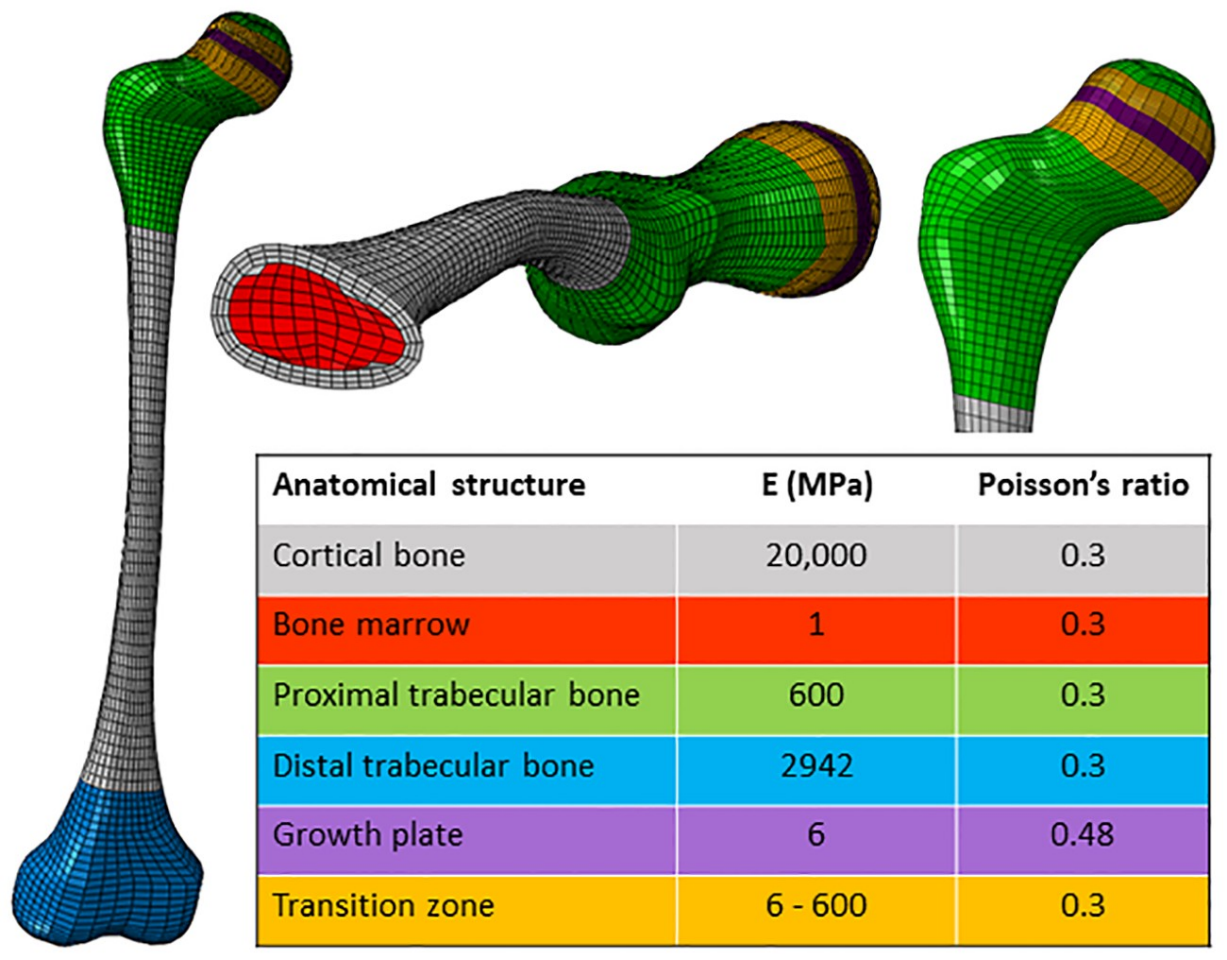
Material properties of the FE model. E = modulus of elasticity.

Fig 1 provides an overview of the FE workflow. MRI images were collected (FE1) and segmented in Mimics (FE2) (Materialise, Leuven, Belgium). From the segmented surface mesh, ANSA (BETA CAE Systems, Root Switzerland) was used to create a hexahedral mesh of the femur with 22,560 elements, including rows of elements representing the growth plate (FE3). The mesh was exported as an Abaqus (Simulia, UK) input file (FE4). This file was imported to Mimics (Materialise, Leuven, Belgium) to define material properties based on the masks created during the segmentation. Material properties (Fig 2) were chosen to be elastic, isotropic and homogenous, similar to previous studies [15,17]. Seven rows of elements were used to model the growth plate and ten rows of elements above and below the growth plate formed a transition zone with a linearly decreasing elastic modulus from the trabecular bone to the growth plate to represent the mineralizing bone tissue. The final FE model of the femur can be downloaded from https://simtk.org/projects/normal-load.

**Fig 3:**
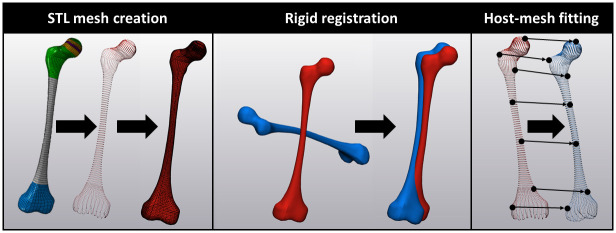
Schematic illustration of the morphing procedure. First, a surface STL mesh was created from the FE model. Afterwards, a rigid registration was utilised to ensure the target (surface of desired femur model) and source (surface of FE model) were crudely aligned. Finally, a host-mesh fitting protocol was implemented to morph the source points to the target points.

Finally, the FE model from the MRI images was morphed to match the geometry of the musculoskeletal models (FE7), therefore ensuring consistency between the musculoskeletal and FE models. To this end, the reference geometry from the SIMM model was converted to a surface mesh (FE5) (stereolithography (STL) file). This surface mesh was modified with a customized Matlab script to match the user-defined NSA and AVA of the musculoskeletal models described above (FE6). This step was needed because SIMM does not create altered geometry files for the modified models (Table 1). Morphing (Fig 3) was done within Python using an open-source package [32]. First, the surface points from the FE model were extracted, and a surface STL mesh created. The surface points from the FE model (source) were then morphed to match the morphed reference geometry STL (target). This was done in two steps. First, a rigid registration was utilised to ensure the target and source were crudely aligned. Second, a host-mesh fitting protocol was implemented to non-rigidly morph the source points to the target points, resulting in a highly accurate fit between the two models. The transformation applied during both, registration and host-mesh fitting, were applied to a set of internal passive points from the FE model. Following morphing, the morphed surface, and morphed passive points were re-assembled into a FE model containing both the surface and internal points representing the morphed geometries. For all morphing of the FE bone to the desired SIMM bone model geometry, average root mean squared differences across the entire surface of the femur was below 3 mm (mean ± standard deviation 2.1 ± 0.6 mm). Furthermore, visual inspection and comparison of the FE bone model and surface mesh of the bone model showed correspondence in geometry, and most importantly NSA and AVA angle, which was crucial for our investigation (S1 Fig in S1 File). In some models, the morphing led to elements with a negative volume. This, however, was only the case for a maximum of two elements per model (0.009% of all elements), which were distal to the growth plate (see S2 Fig in S1 File in the electronic appendix). Hence, if negative elements were present, we removed these elements from the FE model to enable successful simulations. This had no impact on the growth simulations.

### Mechanobiological growth simulations

**Fig 4:**
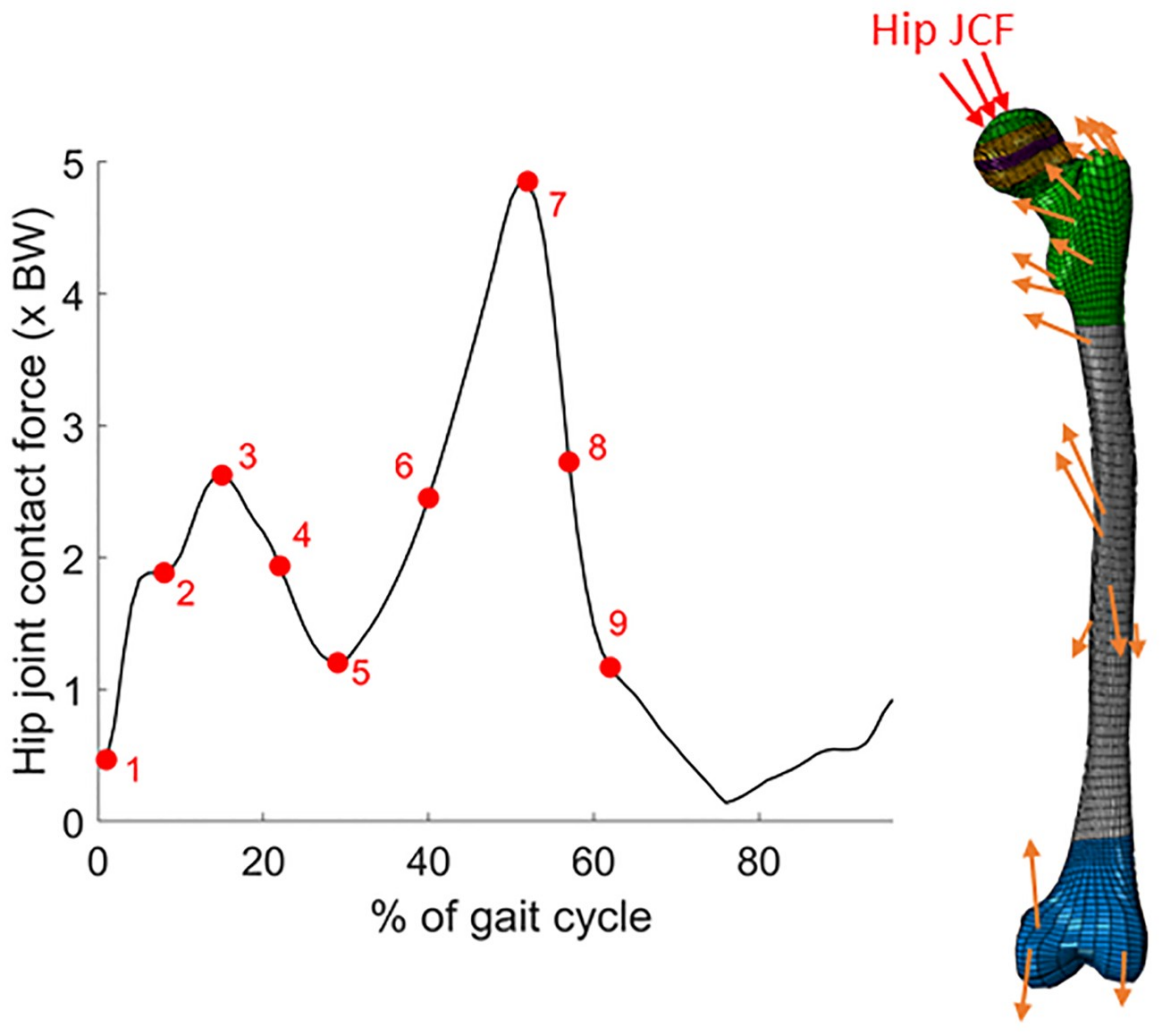
Left: Resultant hip joint contact force (JCF), in which the nine load instances (red dots) used for the FE analysis are highlighted. BW = body weight. Right: FE model with a schematic illustration of the applied loads. Additionally to the hip JCF (red arrows), the following muscle forces (orange arrows) were considered during the FE analysis: gluteus maximus, medius and minimus; adductor longus, brevis and magnus; pectineus; iliacus; psoas; quadratus femoris; gemellus; piriformis; biceps femoris; vastus medius, lateralis and intermedius; and the medial and lateral gastrocnemius.

All FE analysis were performed in Abaqus (2017, Simulia, UK). A combination of Python and Matlab scripts were used for implementing the mechanobiological growth workflow. During the FE analysis, femoral condyles positions were fixed in all models. Using hip JCF waveforms from the musculoskeletal simulations, nine sequential load instances were defined similar to Yadav et al. [17] (Fig 4). Each muscle force was applied as a concentrated force at the node closest to the point of insertion projected on the FE model. The muscle attachment and muscle lines of action were obtained from a previously developed OpenSim plugin [33]. Hip JCFs were distributed over a ~30 mm^2^ area nearest the hip JCF’s line of action [15,17].

Femoral growth rate and direction computation was based on a previously developed workflow [15], which assumed that cyclic octahedral shear stress promotes but cyclic hydrostatic compressive stress inhibits growth [34]. Growth rate ($\dot{\varepsilon }$) was calculated as the sum of a biological (${\dot{\varepsilon }}_{b}$) and a mechanical component (${\dot{\varepsilon }}_{m}$):

$$\dot{\varepsilon }={\dot{\varepsilon }}_{b}+{\dot{\varepsilon }}_{m}$$ (1)

Biological growth rate caused by intrinsic genetic and hormonal regulations was assumed to be constant. Hence, the growth potential was only determined by the mechanical component, defined by the osteogenic index (OI):

$${\dot{\varepsilon }}_{m}\approx OI=a∙max\sigma_{Si}+b∙min\sigma_{Hi}\;i=1\ldots 9$$ (2)

where *i* indicated the 9 load instances. *σ*_*S*_ and *σ*_*H*_ were the octahedral shear stress and hydrostatic compressive stress, respectively. *σ*_*S*_ and *σ*_*H*_ were calculated for the distal layer of the proximal growth plate based on the principal stresses (*σ*_1_, *σ*_2_, *σ*_3_) obtained from the FE analysis.

$$\sigma_{s}=\frac{\sqrt{{\left(\sigma_{1}-\sigma_{2}\right)}^{2}+{\left(\sigma_{2}-\sigma_{3}\right)}^{2}+{\left(\sigma_{3}-\sigma_{1}\right)}^{2}}}{3}$$ (3)

$$\sigma_{H}=\frac{\sigma_{1}+\sigma_{2}+\sigma_{3}}{3}$$ (4)

*maxσ*_*Si*_ and *minσ*_*Hi*_ in Eq 2 referred to the maximum *σ*_*S*_ and minimum *σ*_*H*_ obtained from all nine load instances, indicated with *i*. *a* and *b* are constants and determine the relative influence of the octahedral shear and hydrostatic stress. A ratio *b*/*a* of 0.5 was chosen based on the available data from the literature [35–37] and consistent with previous studies [15,16].

Two methods to define the growth direction were proposed in the past. Carriero et al. [15] modelled femoral growth in the direction of the average deformation of the neck, whereas Yadav et al. [16] proposed to model femoral growth in the maximum principal stress direction. Based on a pilot study in which we compared both approaches using simplified load scenarios (see S1 File), proximal femoral growth was modelled in the direction of the average deformation of the neck. Hence, in the same way as in previous studies [15,16], we calculated the growth direction as follows:

$${\hat{GD}}_{FND}=\frac{{\vec{GD}}_{FND}}{\left|{\vec{GD}}_{FND}\right|}$$ (5)

Growth direction was defined by the unit vector ${\hat{GD}}_{FND}.\;{\vec{GD}}_{FND}$ was the vector connecting the base of the femoral neck (NB) with the centre of the femoral head (HC) during the average deflection caused by the nine load instances.

$${\vec{GD}}_{FND}=[{x_{dHC}-x_{dNB}\;,\;y}_{dHC}-y_{dNB}\;,\;z_{dHC}-z_{dNB}]$$ (6)

$$\left[x_{dHC}\;y_{dHC}\;z_{dHC}]=[x_{HC}\;y_{HC}\;z_{HC}]+\frac{1}{9}∙[\sum_{i=1}^{9}d_{HCx}\;\sum_{i=1}^{9}d_{HCy}\;\sum_{i=1}^{9}d_{HCz}\right]$$ (7)

$$\left[x_{dNB}\;y_{dNB}\;z_{dNB}]=[x_{NB}\;y_{NB}\;z_{NB}]+\frac{1}{9}∙[\sum_{i=1}^{9}d_{NBx}\;\sum_{i=1}^{9}d_{NBy}\;\sum_{i=1}^{9}d_{NBz}\right]$$ (8)

[*x*_*HC*_, *y*_*HC*_, *z*_*HC*_] and [*x*_*NB*_, *y*_*NB*_, *z*_*NB*_] were the original coordinates of HC and NB. [*d*_*HCx*_, *d*_*HCy*_, *d*_*HCz*_] and [*d*_*NBx*_, *d*_*NBy*_, *d*_*NBz*_] were the deflections of HC and NB. A coordinate system for each element of the growth region was defined based on ${\hat{GD}}_{FND}$. In a second FE analysis, orthonormal thermal expansion was used to simulate bone growth. The coefficient of thermal expansion was defined as one in x-direction (${\hat{GD}}_{FND}$ direction) and zero in the remaining two directions. The specific growth rate for each element (Eq 1) was applied as temperature loads. Afterwards, nodal coordinates of the whole femur were updated.

$$[n_{{Gx}_{i}}\;n_{Gy_{i}}\;n_{Gz_{i}}]=[n_{x_{i}}\;n_{y_{i}}\;n_{z_{i}}]+[d_{x_{i}}\;d_{y_{i}}\;d_{z_{i}}]∙10\;i=1\;\ldots \;25,143$$ (9)

$[n_{x_{i}}\;n_{y_{i}}\;n_{z_{i}}]\;$ were the original nodal coordinates, $\left[d_{x_{i}}\;d_{y_{i}}\;d_{z_{i}}\right]$ were the displacement caused by the growth simulation, $\left[n_{{Gx}_{i}}\;n_{Gy_{i}}\;n_{Gz_{i}}\right]$ were the updated nodal coordinates after the growth simulations, and *i* indicated the nodes. To see a clear impact of the different geometries on the growth simulations without the need to model femoral growth over several layers of the growth plate, we multiplied the observed displacement by a constant factor of 10.

### Data analysis

**Fig 5:**
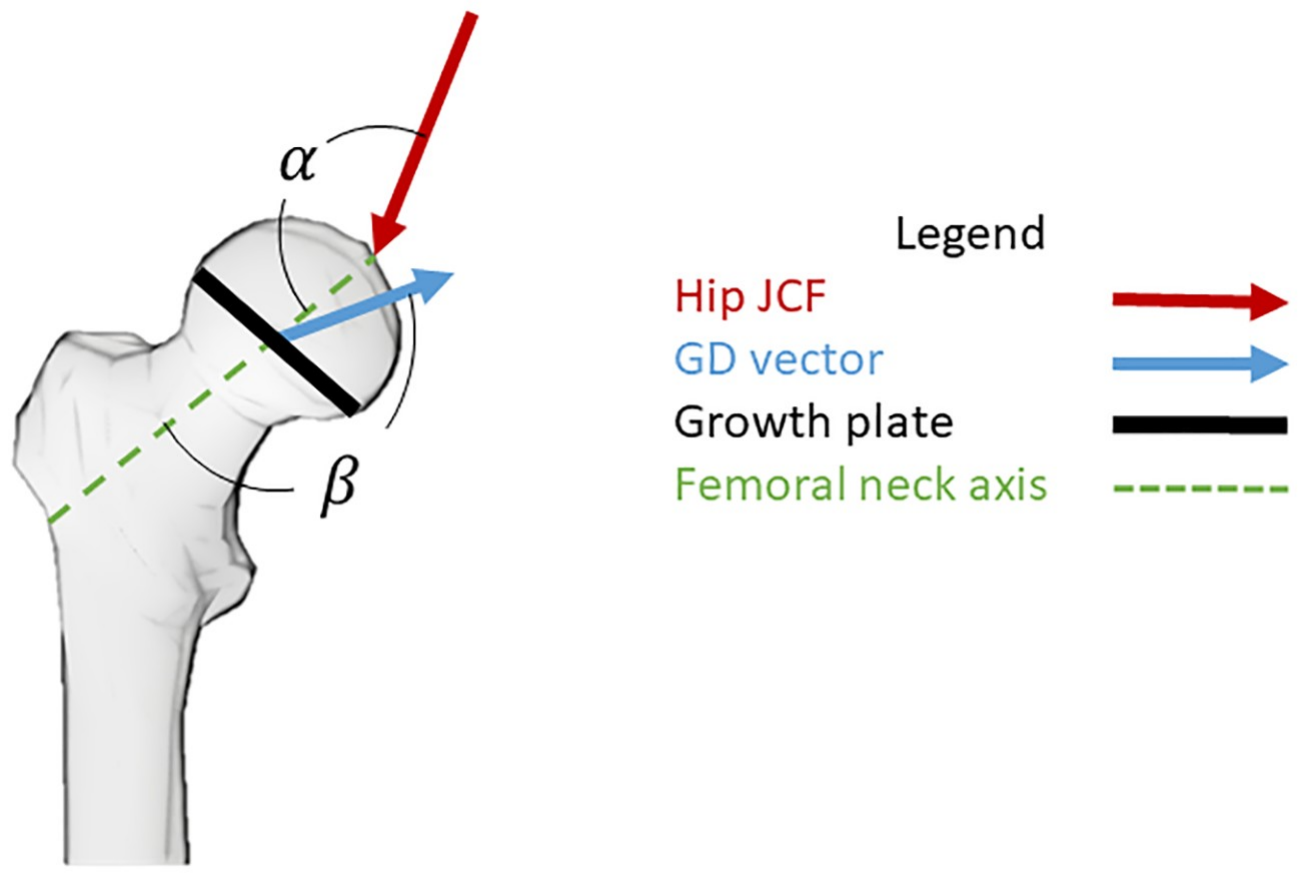
Schematic illustration of the angle between the hip joint contact force (JCF) and femoral neck axis (α) and the angle between the growth direction (GD) vector and the femoral neck axis (β). These angles were analysed to get a better understanding about the impact of the femoral geometry on hip JCF and growth simulations.

Root-mean-square-differences (RMSD) were used to compare hip JCF waveforms between the reference model and the musculoskeletal models with systematically altered NSA and AVA. For the mechanobiological growth simulations, changes in femoral NSA and AVA between the original and ‘grown’ model were calculated using a customized Matlab code (described in the electronic appendix) and compared between different models. Furthermore, we compared the average orientation of the hip JCF and the growth direction vector in reference to the femoral neck axis between the different models (Fig 5).

## Results

**Fig 6:**
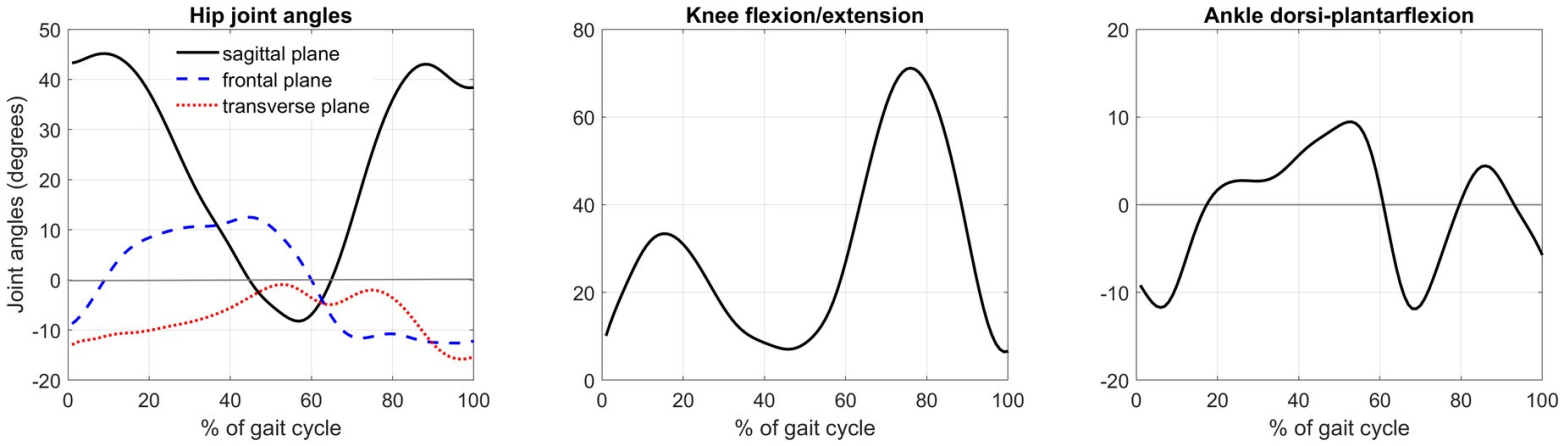
Hip, knee and ankle joint kinematics from our participant TD01.

Our participant walked with an average walking velocity of 1.4 m/s. Lower limb joint kinematics (Fig 6) were comparable to previous investigations [38].

### Hip JCF

**Fig 7:**
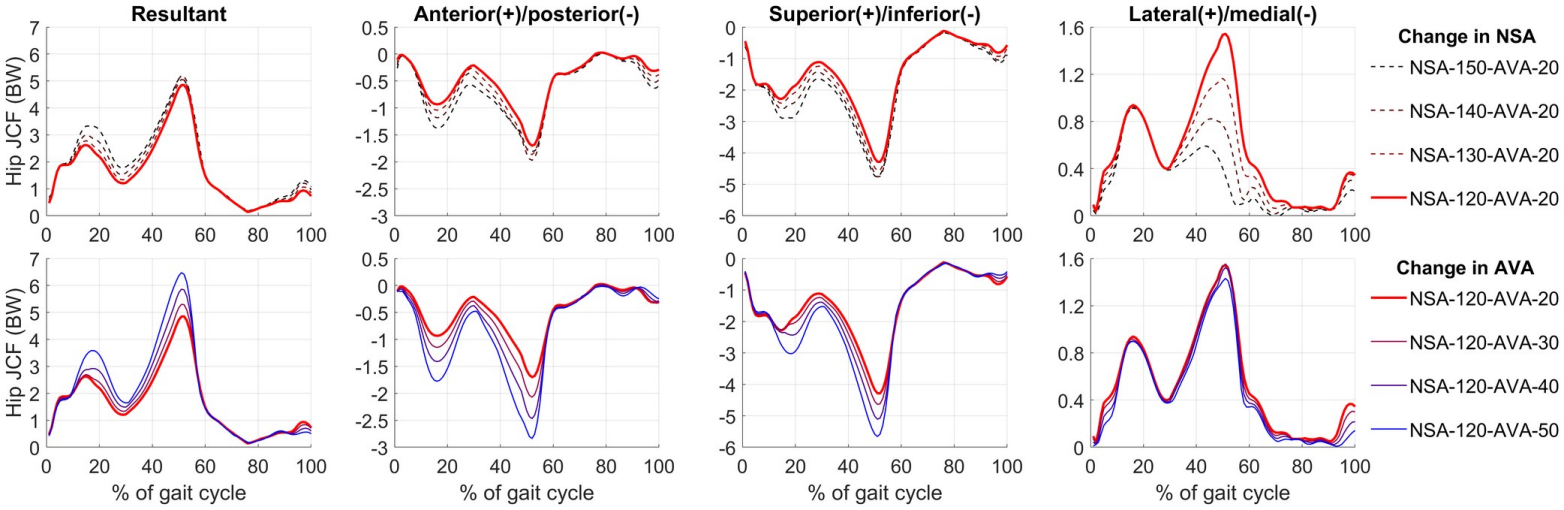
Hip joint contact forces (JCF): Resultant and for each anatomical direction. BW = body weight. The first row shows the impact of different neck-shaft angle (NSA) on hip JCF (dashed waveforms). The second row shows the impact of different anteversion angles (AVA) on hip JCF (solid waveforms).

**Fig 8:**
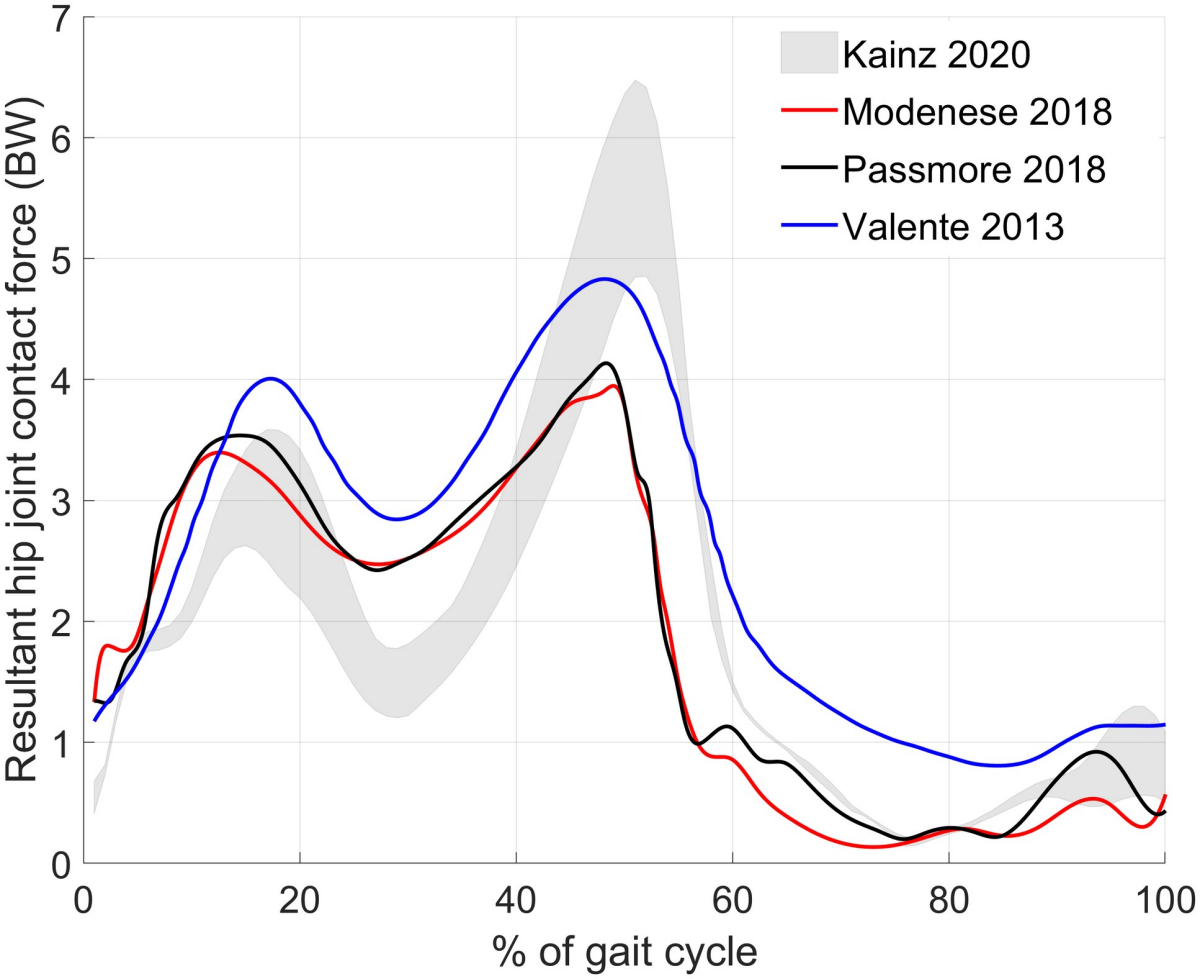
Mean resultant hip joint contact forces from previous studies (red from [39], black from [19], and blue from [40]) compared to our results (grey shaded area).

Hip JCF (Figs 7 and 8) were comparable to previous studies [19,39,40]. Compared to the generic geometry (NSA-120-AVA-20), increasing the AVA and NSA increased hip JCF. RMSD between the reference model and the models with altered AVA were 0.05 body weight (BW), 0.10 BW and 0.17 BW for the models with 30°, 40° and 50° of AVA, respectively. RMSD between the reference model and the models with altered NSA were 0.02 BW, 0.05 BW and 0.10 BW for the models with 130°, 140° and 150° of NSA, respectively. Increasing the NSA primarily increased the first peak of the hip JCF, whereas increasing the AVA increased both peaks of the hip JCF and had a larger impact on average hip JCF.

**Fig 9:**
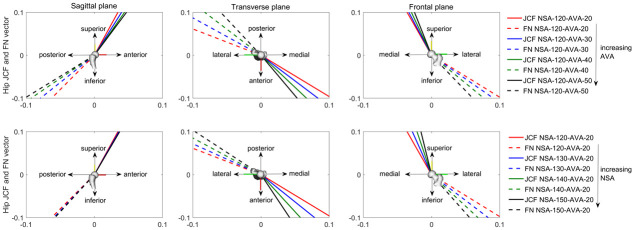
Hip joint contact forces (JCF, solid lines) and femoral neck (FN, dashed lines) orientation for each anatomical plane expressed in the femoral segment coordinate system from the musculoskeletal OpenSim model. For the hip JCF, the average orientation from all nine considered load instances (Fig 4) are visualized. First row shows the orientations for models with increasing anteversion angle (AVA). Second row shows the orientations for models with increasing neck-shaft angle (NSA). The hip JCF generally aligned with the orientation of the FN.

The orientation of the hip JCF changed with the altered geometry. Increasing NSA changed the orientation of the hip JCF to a more vertical direction, whereas increasing AVA led to a more posterior direction (Fig 9). Interestingly, the relative angle between the hip JCF and femoral neck axis only slightly changed (Fig 12).

### Proximal femoral growth simulations

**Fig 10:**
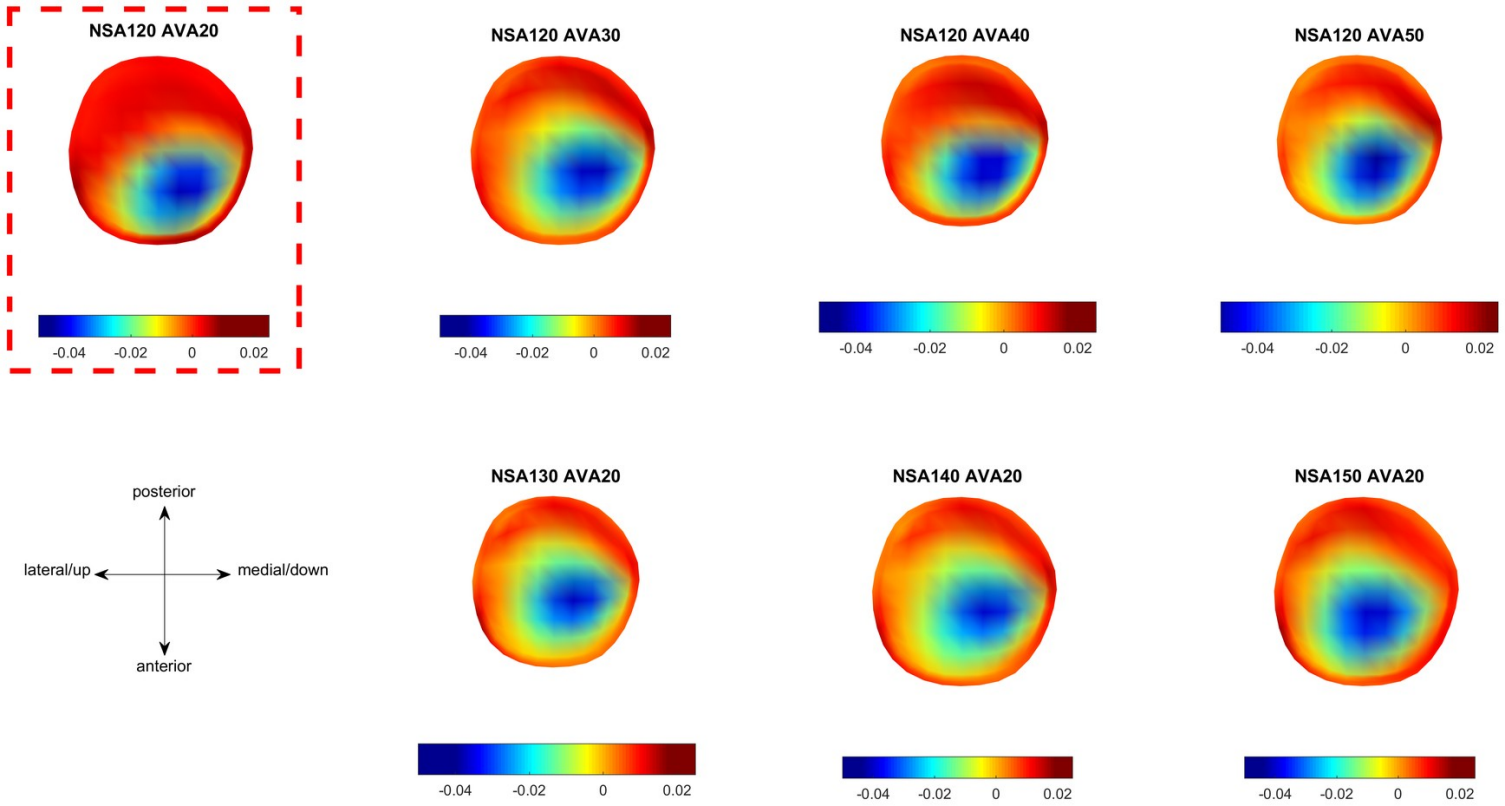
Osteogenic index distribution from the models with different femoral geometries. First row: altered anteversion angle (AVA). Second row: altered neck-shaft angle (NSA). The reference model is highlighted with the red, dashed box.

The osteogenic index was similar between all analysed models (Fig 10). Comparable to previous studies in typically developing children, the osteogenic index was higher in the lateral and posterior regions [15,17].

**Fig 11:**
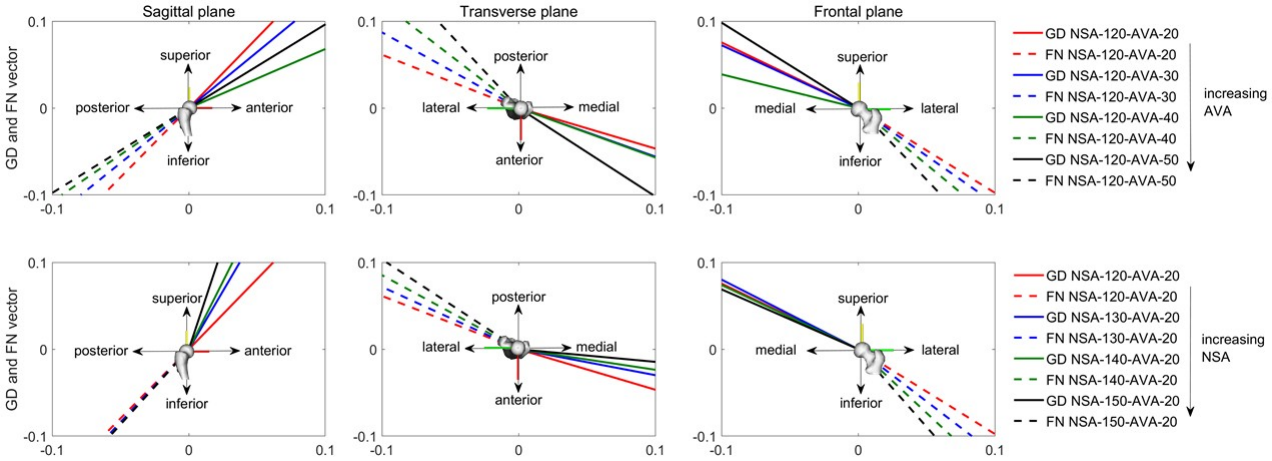
Growth direction (GD, solid lines) and femoral neck (FN, dashed lines) orientation for each anatomical plane expressed in the femoral segment coordinate system from the musculoskeletal OpenSim model. First row shows the orientations for models with increasing anteversion angle (AVA). Second row shows the orientations for models with increasing neck-shaft angle (NSA).

**Fig 12:**
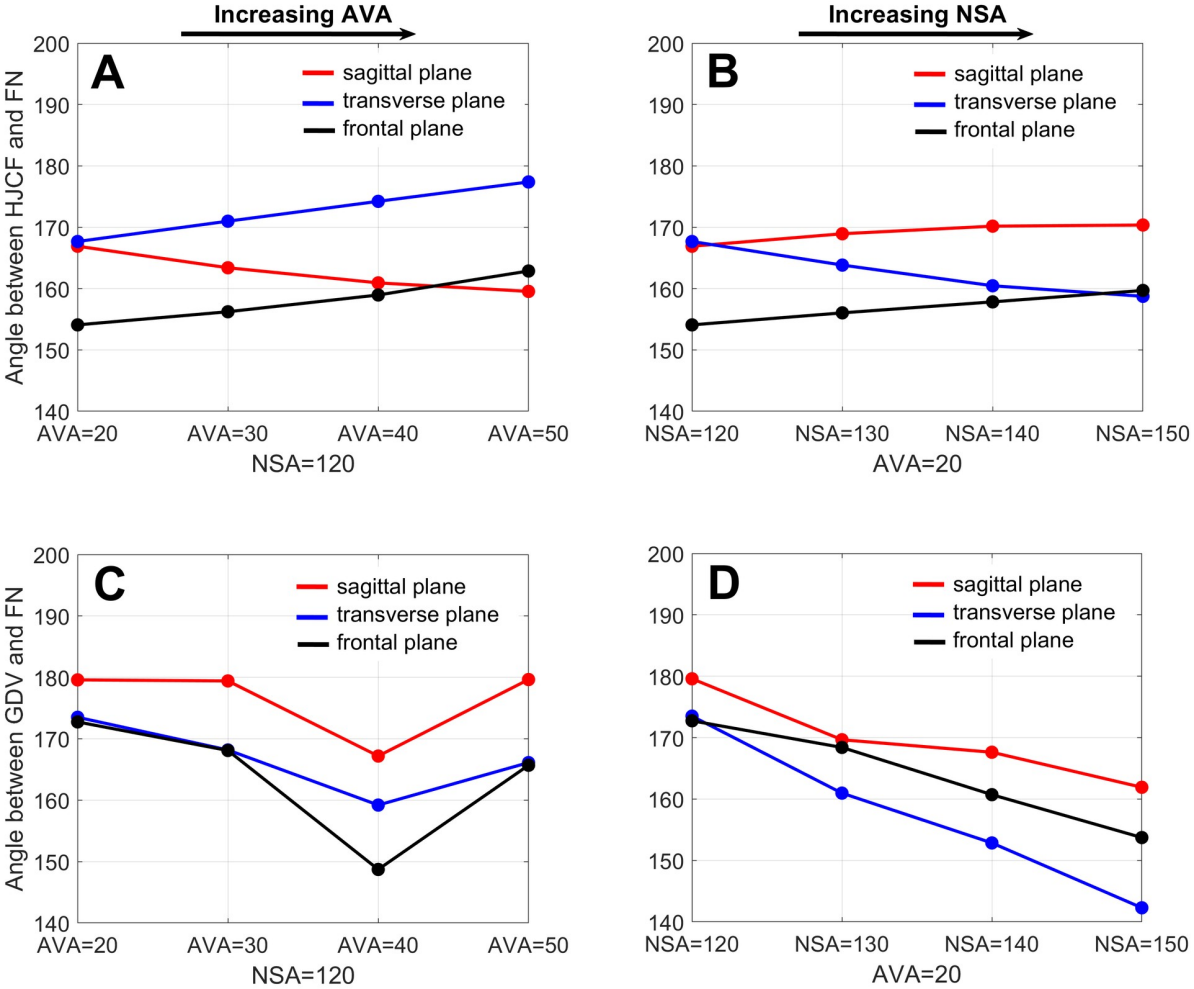
Summary of angles from Figs 9 and 11. First row: Relative angle between the hip joint contact force (HJCF) and femoral neck axis (FN) for the models with increasing anteversion angle (A) and increasing neck-shaft angle (B). Second row: Relative angle between the growth direction vector (GDV) and femoral neck axis (FN) for the models with increasing anteversion angle (C) and increasing neck-shaft angle (D).

Growth direction changed with the altered geometry. Increasing the AVA led to a more anterior orientated femoral growth, whereas increasing the NSA led to a more superior oriented growth direction (Fig 11). The relative angle between the growth direction vector and femoral neck axis decreased with increasing AVA and NSA with maximum differences in the sagittal, transverse and frontal plane of 12°, 14° and 24° for altered AVA and 18°, 31° and 19° for altered NSA (Fig 12).

**Fig 13:**
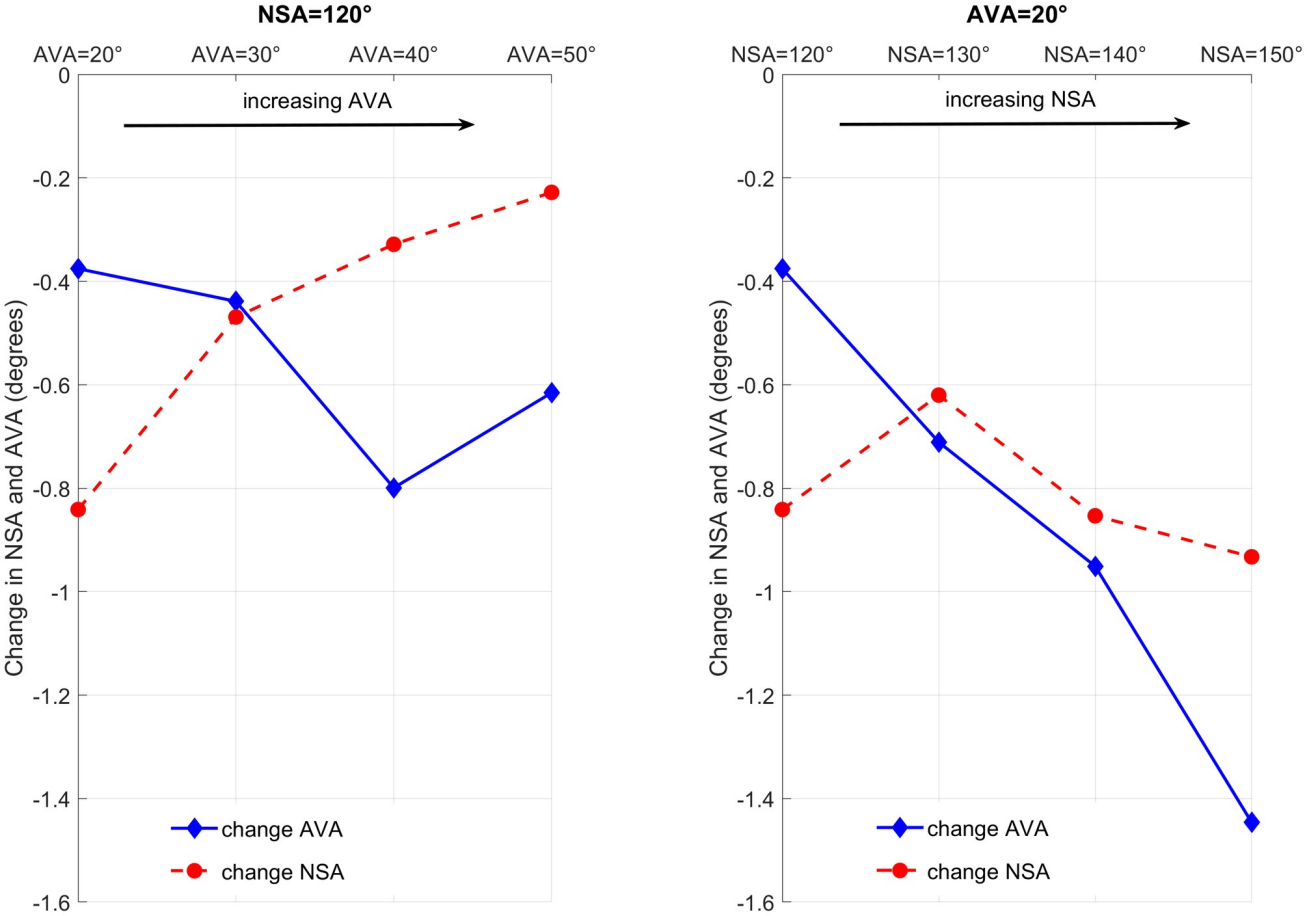
Results from the femoral growth simulations, i.e. changes in neck-shaft angle (NSA) and anteversion angle (AVA), based on the different musculoskeletal and FE models.

In all analysed models, NSA and AVA decreased due to the growth simulations (Fig 13), in agreement with the expected changes of the femoral geometry in growing children [7,27]. Increasing the AVA in our models from 20° to 50° decreased changes in NSA from -0.84° to -0.23° and increased changes in AVA from -0.38° to -0.62°. Increasing the NSA in our models from 120° to 150° increased changes in AVA from -0.38° to -1.45° but only had a small impact on changes in NSA (slightly increased change from -0.84° to -0.93°).

## Discussion

The aim of this study was to investigate the impact of systematic variations in femoral geometry on hip JCF and proximal femoral growth. Our findings showed that hip JCF magnitude and orientation change with altered femoral NSA and AVA. Interestingly, the hip JCF orientation followed the femoral neck axis, e.g. a more anterior oriented neck axis led to a more posterior oriented hip JCF, which resulted in a relative constant angle between the neck axis and hip JCF. Growth simulations showed that femoral geometry influences the prediction of proximal femoral growth, although the osteogenic index, indicative of the overall growth rate, showed only minimal changes with altered NSA and AVA. Hence, the altered growth directions, due to changes in the average deformation direction under the different load cases, was the primary cause of the observed changes in femoral growth predictions.

Hip JCF of our participant were directed inferior, lateral and posterior, which was in agreement with previous studies [41,42]. The magnitude of hip JCF (maximum of 4.9 BW for the reference model with a NSA of 120° and AVA of 20°) was higher than observed in instrumented hip implants of elderly people (maximum of 2.9 BW) [41] but in agreement with a previous modelling study of children (mean ± standard deviation of peak hip JCF of 4.0 ± 0.9 BW) [39]. Differences in leg lengths and consequently step lengths and/or cadence between children and elderly people may explain the increased hip JCF in children compared to elderly people.

Changes in hip JCF due to the altered geometry were in agreement with our hypothesis (increased NSA and AVA lead to increased hip JCF) and with previous investigations. Passmore et al. [19] calculated hip JCF in patients with increased femoral AVA and compared their results with a model with unchanged AVA. The authors found that increased AVA increases both peaks of the hip JCF, which agrees with our findings. Furthermore, we showed that, compared to increasing the AVA, increasing the NSA only slightly increases the anterior-posterior and vertical component of the hip JCF, which is in agreement with a study from Lenaerts et al. [20]. To the best of the authors’ knowledge, however, this is the first study, which showed that the orientation of the hip JCF follows the proximal femoral geometry, i.e. orientation of the femoral neck axis.

Previous studies only compared the osteogenic index between different participants with different walking patterns and/or femoral geometries [15,17]. Hence, from these studies it was not possible to conclude if different NSA and AVA influence the osteogenic index. Our growth simulations showed that the osteogenic index barely changes with altered NSA and AVA. This is likely due to the observed fact that the orientation of the hip JCF in reference to the femoral neck axis, and therefore also in reference to the growth plate, remains relatively constant (Figs 9 and 12), leading to similar principal stresses in the elements of the growth plate.

The predicted growth direction changed with increased NSA and AVA and, therefore, led to different femoral growth prediction between our analysed models. Altering the femoral geometry changed the hip JCF orientation and therefore the hip JCF’s lever-arm relative to the constraints of the FE model (femoral condyles). These altered loading condition had an impact on the deformation of the model. Hence, although the stresses at the growth plate did not change a lot, the altered geometry and loading conditions changed the deflection of the femoral neck and therefore had an impact on the calculation of the growth direction.

A core principle in mechanobiology is that altered loading conditions (e.g. altered hip JCF) modulate skeletal growth [43]. In the models with increasing NSA the angle between the hip JCF and femoral neck axis decreased in the transverse plane (Fig 12B), leading to a more posterior oriented hip JCF. Based on the presumed mechanobiological response, posterior oriented hip JCF should lead to decreased AVA, which was confirmed by our growth simulations (Fig 13). Contrary, in the models with increasing AVA the angle between the hip JCF and femoral neck axis increased in the transverse plane (Fig 12A), leading to a more lateral oriented hip JCF. Lateral oriented forces counteract a decrease in NSA and, therefore, we would expect a reduction in changes of the NSA, which was also confirmed by our growth simulations (NSA decreased less in the models with increasing AVA, Fig 13). Hence, it seems that our growth simulations agree with presumed mechanobiological responses and predict the expected changes based on altered loading conditions.

We modelled bone growth in the direction of the average deformation of the femoral neck and found decreasing NSA and AVA in all our models. This is in agreement with the expected changes of the femoral geometry in growing children [7,27] but contrary to the modelling study from Carriero et al. [15] and Yadav et al. [16]. Carriero et al. [15] found a decrease in NSA but an increase in AVA in a typically developing child. Their model was created without the use of medical images and, therefore, included a very simplified geometry based on the shape of an adult femur. These simplifications might be the reasons for the different growth results between Carriero et al. [15] and our study. Yadav et al. [16] used a medical imaging-based FE model and found decreasing NSA and increasing AVA when modelling femoral growth in the direction of the average neck deformation. Differences in femoral geometry, growth plate shape and location, and hip JCF between Yadav et al. [16] and our study are likely the reason for the observed differences in the prediction of femoral growth.

In this study the hip JCF were only calculated for one typically developing child. In children with pathological walking patterns, e.g. crouch gait, increasing the NSA and AVA might influence hip JCF in a different way. Furthermore, evaluating if extreme NSA and AVA alter gait kinematics and therefore the hip JCF in typically developing children was above the scope of this study and should be investigate in the future. We based our FE models on MRI of one child and morphed this model to adjust NSA and AVA. Hence, we did not account for variations of the internal structure of the femur (e.g. shape or orientation of the growth plate), which might have influenced the osteogenic index and femoral growth predictions. However, we assume that the impact of altered NSA and AVA on femoral growth predictions would follow a similar trend in FE models based on different participants. We used linear elastic, isotropic, and homogeneous material properties, which greatly simplifies poro-viscoelastic inhomogeneous anisotropic properties of both bone and cartilage. However, with short loading durations and macroscopic (whole organ) viewpoint, these simplifications are adequate for studying the mechanobiology of cartilage based on physiological loads [44,45]. Furthermore, the chosen number of load scenarios, the chosen growth direction and chosen constant parameters in the growth algorithm (${\dot{\varepsilon }}_{b},\;a,\;b$) might have influenced our findings. We, however, were mainly interested in the relative behaviour of the models, rather than the exact magnitudes and, therefore, these modelling assumptions seemed to be adequate for the purpose of our study. Nevertheless, it would be worthwhile to investigate the impact of different and alternative parameters (e.g. strain-based measures) on simulation results in future studies. We only modelled femoral growth at the proximal growth plate and did not consider growth at distal epiphysis, greater trochanter and lesser trochanter, nor did we model periosteal ossification (growth in width). This simplification was adequate for the purpose of this study but might not be valid for an accurate prediction of femoral growth of an individual. Future research based on medical images collected from children on two occasions (e.g. 2 years apart) is needed to access the accuracy of the growth simulation workflow and validate some of the modelling assumptions.

## Conclusion

Our findings indicated that hip JCF increase with increasing NSA and AVA when the kinematics are maintained. Furthermore, the orientation of the hip JCF followed the orientation of the femoral neck axis. Consequently, the osteogenic index barely changed with altered NSA and AVA. Nevertheless, femoral growth predictions were sensitive to the femoral geometry due to changes in the predicted growth directions. Altered NSA had a bigger impact on the growth results than the altered AVA. Our findings enable to estimate the uncertainties associated with growth simulations based on generic FE models (e.g. NSA of 120° and AVA of 20°), which is essential for moving towards more clinically relevant research questions.

## Supporting information

S1 File (PDF)
