# Supplementary material for: A multi-scale modelling framework combining musculoskeletal rigid-body simulations with adaptive finite element analyses, to evaluate the impact of femoral geometry on hip joint contact forces and femoral bone growth
Source: PLoS One. 2020 Jul 23;15(7):e0235966. doi: 10.1371/journal.pone.0235966 (PMC7377390; doi:10.1371/journal.pone.0235966)
Supplement: S1 File — (PDF) [file pone.0235966.s001.pdf]

SUPPLEMENTARY FIGURES/MATERIAL

Supplementary figures

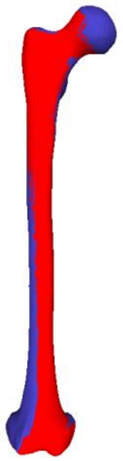

Fig. S1. Example for the correspondence between the morphed FE bone (red) and the desired SIMM bone (blue).

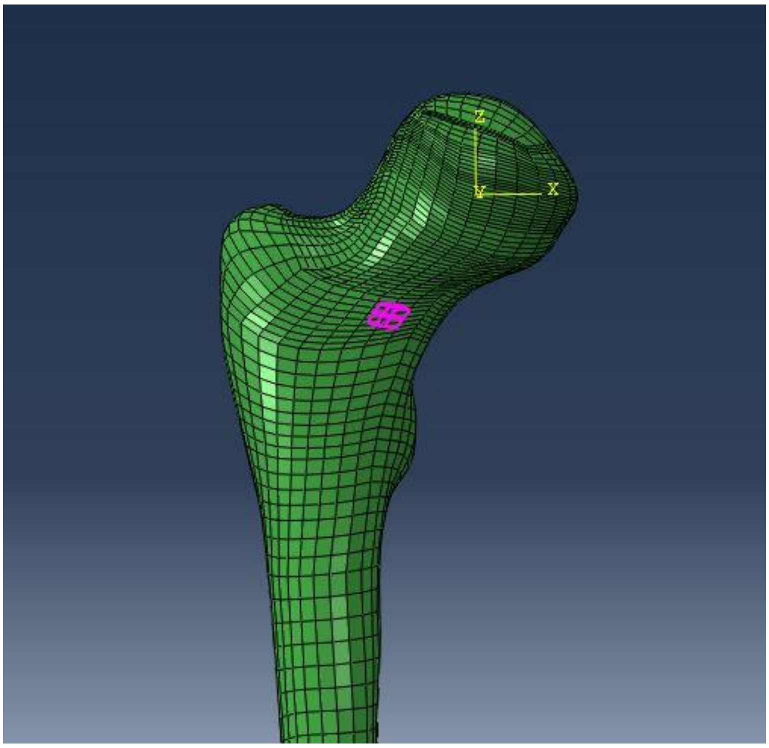

Fig. S2. Example of an FE model, which highlights the elements with negative volume. In some models, the morphing led to elements with a negative volume. This, however, was only the case for a maximum of two elements per model (0.009% of all elements), which were distal to the growth plate as highlighted in this figure.

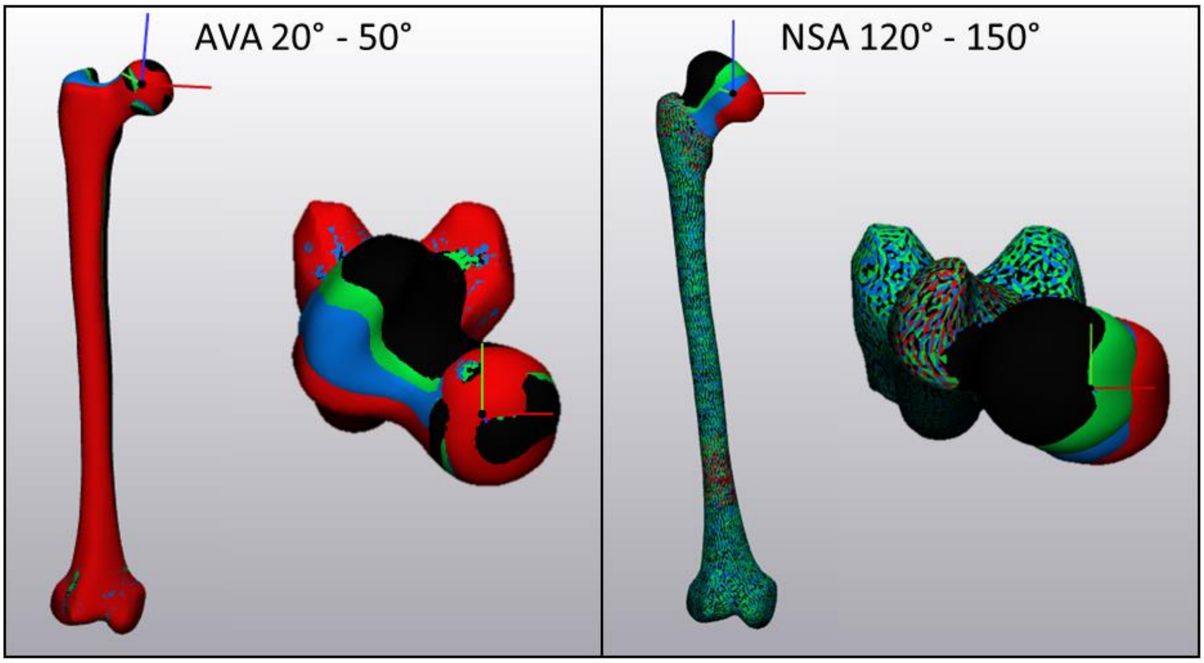

Fig. S3. Femur models with different anteversion angles (AVA) and neck-shaft angles (NSA). The models with different NSA were scaled to the reference length of the femur prior to the morphing. The reference model (NSA 120° and AVA 20°) is shown in red. Models with a NSA of 130°, 140° and 150° are shown in blue, green and black color, respectively. Similar, models with a AVA of 30°, 40° and 50° are shown in blue, green and black color, respectively.

Supplementary analysis

Evaluation of previously proposed methods to define the proximal femoral growth direction: Average neck deflection versus maximum principal stress direction

Introduction

Up until now, only a small number of studies used a multi-scale adaptive modelling approach to predict proximal femoral growth trends in children (Carriero et al., 2011; Yadav et al., 2017, 2016). In these studies, growth has been simulated either in the direction of the average deflection of the femoral neck (avgDef) (Carriero et al., 2011) or the direction of the maximum principal stresses (maxPSD) (Yadav et al., 2017, 2016).

Carriero et al. (2011) proposed the avgDef approach and found a decrease in neck-shaft angle (NSA) and slight increase in anteversion angle (AVA) when modelling femoral growth in one typically developing child. Their study, however, included a musculoskeletal model and adaptive finite element model based on a generic adult model and, therefore, did not consider age- or subject-specific musculoskeletal geometry. Yadav et al. (2016) proposed to model proximal femoral growth in the maxPSD and found a decrease in NSA and AVA when using a FE model based on medical images of one child. In this study, however, the material properties for the proximal trabecular bone were chosen to be only 58 MPa, which is unrealistically small (Carter and Hazes, 1977).

To the best of the authors' knowledge, no studies tried to validate the growth direction assumptions in a simplified loading scenario with known growth trends so far. Hence, to select the most appropriate method for the main manuscript we investigated if both growth direction approaches lead to reasonable results in a simplified loading scenario.

Methods

The development and details of the finite element model and the mechanobiological bone growth workflow is described in the main manuscript. The reference model with a NSA of 120 degrees and AVA of 20 degrees was used for this investigation.

Experimental studies showed that the direction of growth depends on the applied load (Arkin and Katz, 1956). Furthermore, bones do not grow against the direction of the applied load. For example, in healthy individuals the component of the hip joint contact force is the

highest in vertical direction (pointing from superior to inferior) leading to a decrease in NSA from approximately 150 degrees at birth to 120 degrees at skeletal maternity (Bobroff et al., 1999). Based on that, we tested following three load scenarios in which we knew how the femoral geometry should change during the growth modelling process (Figure 1):

- **Only vertical load** (from superior to inferior) on the femoral head, which should **decrease the NSA.**
- **Only lateral load** (from medial to lateral) on the femoral head, which should **increase the NSA.**
- **Only anterior load** (from anterior to posterior) on the femoral head, which should **decrease the AVA.**

The load for each scenario was applied as a constant load (divided over the 9 closest nodes to the force vector on the surface of the femoral head) based on the maximum values of the hip joint contact forces observed in each anatomical direction during normal walking in our participant from the main manuscript. This resulted in a constant load of 1900N, 700N, and 700N, which were applied in vertical, lateral and anterior direction, respectively. No other forces (e.g. muscle forces) were applied to the finite element model.

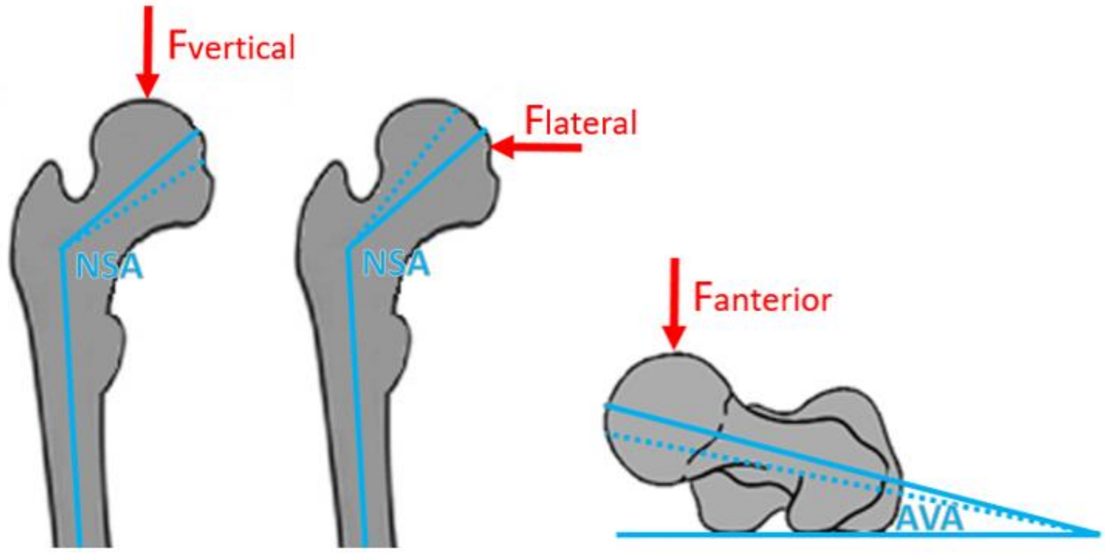

Fig. 1. Schematic illustration of the simplified load scenarios and expected change in neck-shaft angle (NSA) and anteversion angle (AVA). Solid blue lines = original NSA or AVA. Dotted blue lines = expected geometry change due to the simplified load cases.

The osteogenic index was calculated (described in the main manuscript) and used to define the amount of growth for each element in the growth plate. Growth directions were

defined by either the avgDef or maxPSD directions. Detailed description including the mathematical equations for both approaches can be found in Yadav et al. (2016). Growth was simulated for one layer of the growth plate and the changes in NSA and AVA due to the growth simulations were computed for each model (i.e. avgDef and maxPSD) and compared to our expected changes. Similar to the main manuscript, the obtained changes in nodal coordinates were multiplied by 10 to see a clear trend in the change of the NSA and AVA without the need of simulating femoral growth over several layers of the growth plate.

Results and discussion

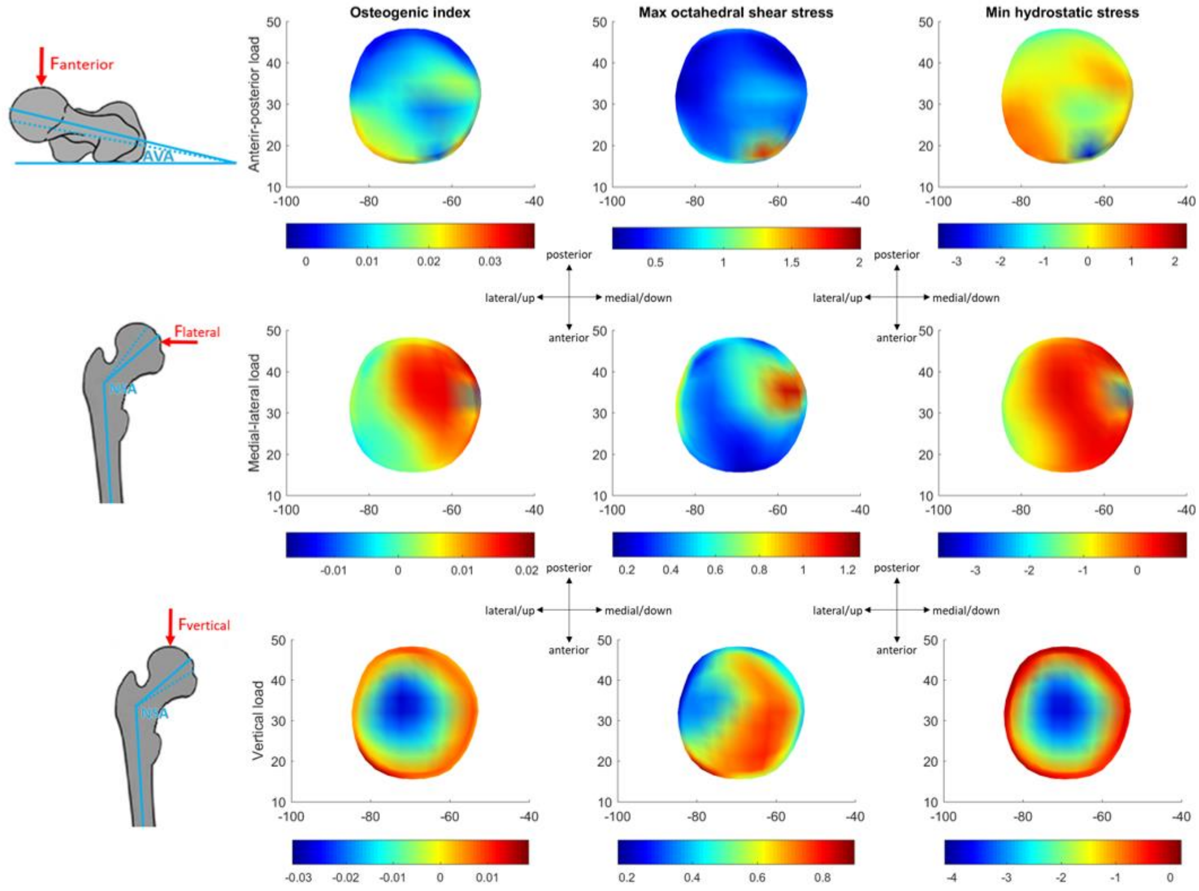

Fig. 2. Osteogenic index (left column), maximum octahedral shear stress (middle column) and minimum hydrostatic stress (left column) obtained with the simplified load cases.

The growth components (maximum octahedral shear stress, minimum hydrostatic stress and osteogenic index) obtained from the simplified loading scenarios are shown in figure 2. Independent of the growth direction approach, these values are in agreement with our understanding of femoral bone growth and our expectations of the osteogenic index. For example, a lateral force was expected to increase the NSA and, therefore, the osteogenic index

should be higher on the medial/lower side of the growth plate than on the lateral side, which is what we observed in our results (Fig. 2). Similar, the anterior force was expected to lead to a higher osteogenic index on the anterior than posterior side to enable a decrease of AVA, which is in agreement with our findings. For the vertical direction, the results showed a bowl shaped osteogenic index, which made the interpretation more difficult and could be due to the shape of the growth plate.

The femoral geometry (NSA and AVA) changed due to the growth simulations in agreement with our expectations when using the avgDef approach (Tab. 1). The maxPSD approach led to reasonable results only for loads in the vertical direction.

Table 1. Change in neck-shaft angle (NSA) and anteversion angle (AVA) for each loading scenario and growth direction method (avgDef and maxPSD). Green bold values are in agreement with the expectation of the growth trend; red bold values are in contrast to the expected growth trends; and black values are neutral changes, which we could not be validated with our loading scenarios. All values are in degrees.

Method	Parameter	Anterior force	Lateral force	Vertical force
avgDef	NSA	-0.006	**+0.008**	**-0.024**
	AVA	**-0.004**	+0.007	-0.001
maxPSD	NSA	-0.013	**-0.032**	**-0.035**
	AVA	**+0.008**	-0.016	-0.004

Conclusion

Our findings showed that only growth simulations in the avgDef direction led to reasonable results in all simplified load scenarios and, therefore, the avgDef direction was used for all simulations in the main manuscript.

References

Arkin, A.M., Katz, J.F., 1956. The effects of pressure on epiphyseal growth; the mechanism of plasticity of growing bone. J. Bone Joint Surg. Am. 38–A, 1056–76.

Bobroff, E.D., Chambers, H.G., Sartoris, D.J., Wyatt, M.P., Sutherland, D.H., 1999. Femoral anteversion and neck-shaft angle in children with cerebral palsy. Clin. Orthop. Relat. Res. 194–204.

Carriero, A., Jonkers, I., Shefelbine, S.J., 2011. Mechanobiological prediction of proximal femoral deformities in children with cerebral palsy. Comput. Methods Biomech. Biomed. Engin. 14, 253–262. doi:10.1080/10255841003682505

Carter, D., Hazes, W., 1977. The compressive behavior of bone as a two-phase porous structure. J Bone Jt. Surg Am. 59, 954–962.

Yadav, P., Shefelbine, S.J., Gutierrez-Farewik, E.M., 2016. Effect of growth plate geometry and growth direction on prediction of proximal femoral morphology. J. Biomech. 49, 1613–1619. doi:10.1016/j.jbiomech.2016.03.039

Yadav, P., Shefelbine, S.J., Pontén, E., Gutierrez-Farewik, E.M., 2017. Influence of muscle groups' activation on proximal femoral growth tendency. Biomech. Model. Mechanobiol. 16, 1869–1883. doi:10.1007/s10237-017-0925-3

Supplementary description

Customized Matlab code to obtain the neck-shaft and anteversion angle from the segmented femurs

The morphometric information regarding the analyzed femurs were obtained using following semi-automated procedure:

- The segmented surface of the femur was imported into Matlab. Then, the general orientation of the femur was obtained by generating a convex hull surrounding the femur surface. In the vast majority of cases, the biggest triangle is defined by two point on the posterior surfaces of the epicondyles and one situated at the level of the intertrocantheric crest.
- An iterative closest point (ICP) algorithm is used to fit a sphere, optimizing radius and position, to the most proximal part of the segmented surface, thus leading to the identification of the center of the femoral head (HC).
- Another ICP is performed to fit a template representing the epicondyles to the distal part of the surface, optimizing rotation, translation and a scaling factor. Medial and lateral epicondyles are associated with the fitted template, the axis connecting them is extended until the intersection with the surface under investigation, and the intersection points are then considered as medial and lateral epicondyles (ME, LE).
- Using these points, the reference system of the femur is generated: Z-axis passing through the epicondyles, Y-axis laying on the plane comprising HC. Next, the surface of the femur is sliced along the direction perpendicular to Y.
- To define the axis of the neck, an iterative procedure is applied in which the axis location is adjusted. The moments of inertia for each of the slices are computed. The slice with the biggest ratio between the principal components was selected. This slice is placed at the level of the neck, and its centroid was computed to generate a first guess for the axis, passing through this point an HC. The proximal part of the femur is sliced a first time along this temporary axis. The slice with the shorter perimeter, defining the neck isthmus, is selected and it's centroid is used to update the neck axis estimate. This process is repeated a second time and the isthmus's center is used to define the final neck axis.
- To define the neck-shaft angle (NSA) and anteversion angle (AVA), the main axis of the femur was identified as passing through 1) the interepiconylar saddle point and 2) the centroid of the slide at the height of the smaller trochanter. The small trochanter is identified as the medial point of the surface most distant from the axis fitting the slices of the proximal femur. The saddle point is obtained by first slicing the distal surface perpendicularly to the Z-axis. The most distal point of each slide was selected and the saddle point is defined as the most proximal of these points.
- AVA was obtained projecting the ME-LE axis and the neck axis onto a plane perpendicular to the femur axis and computing the angle between the projections. NSA was the angle between the neck and the main axis.
